# The Role of Magnetic Resonance Imaging for the Diagnosis of Atypical Parkinsonism

**DOI:** 10.3389/fneur.2020.00665

**Published:** 2020-07-17

**Authors:** Lydia Chougar, Nadya Pyatigorskaya, Bertrand Degos, David Grabli, Stéphane Lehéricy

**Affiliations:** ^1^Institut du Cerveau et de la Moelle épinière–ICM, INSERM U 1127, CNRS UMR 7225, Sorbonne Université, UPMC Univ Paris 06, UMRS 1127, CNRS UMR 7225, Paris, France; ^2^ICM, “Movement Investigations and Therapeutics” Team (MOV'IT), Paris, France; ^3^ICM, Centre de NeuroImagerie de Recherche–CENIR, Paris, France; ^4^Service de Neuroradiologie, Hôpital Pitié-Salpêtrière, APHP, Paris, France; ^5^Dynamics and Pathophysiology of Neuronal Networks Team, Center for Interdisciplinary Research in Biology, Collège de France, CNRS UMR7241/INSERM U1050, MemoLife Labex, Paris, France; ^6^Department of Neurology, Avicenne University Hospital, Sorbonne Paris Nord University, Bobigny, France; ^7^Département des Maladies du Système Nerveux, Hôpital Pitié-Salpêtrière, APHP, Paris, France

**Keywords:** parkinson's disease, progressive supranuclear palsy, multiple system atrophy, multimodal magnetic resonance imaging, diagnosis, machine learning

## Abstract

The diagnosis of Parkinson's disease and atypical Parkinsonism remains clinically difficult, especially at the early stage of the disease, since there is a significant overlap of symptoms. Multimodal MRI has significantly improved diagnostic accuracy and understanding of the pathophysiology of Parkinsonian disorders. Structural and quantitative MRI sequences provide biomarkers sensitive to different tissue properties that detect abnormalities specific to each disease and contribute to the diagnosis. Machine learning techniques using these MRI biomarkers can effectively differentiate atypical Parkinsonian syndromes. Such approaches could be implemented in a clinical environment and improve the management of Parkinsonian patients. This review presents different structural and quantitative MRI techniques, their contribution to the differential diagnosis of atypical Parkinsonian disorders and their interest for individual-level diagnosis.

## Introduction

Parkinsonism is defined by the presence of resting tremor, rigidity, bradykinesia, and postural instability. Parkinson's disease (PD) is the most common neurodegenerative cause of Parkinsonism. Atypical Parkinsonism refers to other neurodegenerative disorders that commonly include progressive supranuclear palsy (PSP), corticobasal degeneration, multiple system atrophy (MSA), with its cerebellar (MSA-C) and Parkinsonian (MSA-P) variants, and dementia with Lewy body (1–6). Degeneration of the substantia nigra (SN) is the pathological hallmark of neurodegenerative Parkinsonian disorders (1, 3–7). Neuroimaging plays a major role in the diagnosis of Parkinsonian disorders and the differentiation of PD from atypical Parkinsonism. Magnetic resonance imaging (MRI) improves diagnostic accuracy, reduces the rate of misdiagnosis, facilitates early diagnosis, and may be useful for the follow-up of disease progression (8–11). Usually, the diagnosis of sporadic PD does not require an MRI examination when the clinical presentation is typical. In contrast, MRI is needed when the clinical presentation is atypical, that is, in the presence of symptoms called “red flags,” such as rapid progression of gait impairment, early and recurrent falls and impaired balance, early bulbar or inspiratory respiratory dysfunction, and early and severe autonomic dysfunction (12). In this case, MRI shows a number of features that can help the clinician to distinguish PD from atypical Parkinsonism. This review details the main MRI characteristics of PD and the two main causes of atypical Parkinsonism, PSP and MSA, focusing on the features that can be used in clinical practice.

## MRI Techniques and Biomarkers

MRI provides *in vivo* biomarkers that inform about the underlying neurodegenerative processes. Regional brain atrophy is detected using T1-weighted three-dimensional (3D) sequences and reflects neuronal loss. Diffusion anomalies reflect the presence of microstructural alterations in the tissues, while iron-sensitive imaging detects the presence of iron deposits. Multimodal MRI is defined as the combination of information provided by these different sequences. Brain abnormalities can be assessed in several ways: (i) qualitatively, by visual inspection of regional brain atrophy and signal changes using conventional structural MRI, or (ii) quantitatively, by measurements of changes in volumes, diffusion metrics and iron-related signals (8–10, 13, 14). The main MRI techniques and their respective contributions are summarized in Table 1. The usefulness of neuroimaging biomarkers can be assessed by a five-level scale, as recently proposed for PSP (9). Level 1 defines biomarkers useful at the group level when comparing a specific disease with healthy subjects or other clinically overlapping diseases. Level 2 defines a biomarker useful at the individual level because it reaches a sensitivity and specificity >80% for the clinical diagnosis of a given patient. Level 3 indicates that the biomarker is effective for early clinical diagnosis, when patients present with mild or non-specific symptoms but do not yet meet the clinical criteria for the disease. Level 4 biomarkers are strongly correlated with pathology and could be used as surrogate criteria for pathological diagnosis. Level 5 biomarkers provide a direct measure of the underlying neuropathological changes (9). Here, we detail the different biomarkers in the three diseases in light of this scale.

**Table 1:** Magnetic resonance imaging techniques.

**Techniques**	**Measures**	**Information**
**CONVENTIONAL MRI**		
T1-w,	Shape, volume	Atrophy
T2-w, FLAIR, PD-w	Signal changes	Signal abnormalities (gliosis and demyelination of white matter)
T2*-w, SWI	Iron load	Iron deposition Nigral dorsolateral hyperintensity
Neuromelanin-sensitive sequence	Signal and volume	Content of catecholaminergic neurons in the SNpc and the locus coeruleus
**ASSESSMENT OF REGIONAL ATROPHY**		
3D gradient echo T1-weighted	Anteroposterior midbrain diameter	Midbrain atrophy
	Midbrain to pons midsagittal surface ratio	Ratio of midbrain vs. pons atrophy
	MRPI [(P/M)*(MCP/SCP)]^a^ MRPI 2.0 [MRPI * (V3/VL)]^b^	Brainstem and cerebellar peduncle atrophy
	Automated segmentation	
**IRON-SENSITIVE IMAGING**		
T2*-weighted multiecho Magnitude image	Relaxation times R2*= 1/T2*	Iron load
Magnitude and phase images	Quantitative susceptibility mapping	Iron load
**DIFFUSION IMAGING**		
Diffusion-weighted imaging (DWI)	Apparent diffusion coefficient (ADC), Trace (D)	Magnitude of water diffusion
Diffusion tensor imaging (DTI)	Mean Diffusivity (MD)	Mean magnitude of water diffusion
	Axial Diffusivity (AD) Radial diffusivity (RD)	Magnitude of water diffusion along the main direction Magnitude of water diffusion along the perpendicular direction
	Fractional Anisotropy (FA)	Directionality of water diffusion
Bitensor model	Free water	Magnitude of free water diffusion
Neurite Orientation Dispersion and Density Imaging (NODDI)	Intracellular volume fraction Orientation dispersion index Isotropic volume fraction	Neurite density and dendritic structure
Tractotraphy	Number of tracks, probability of connection	Damage in specific fiber tracts
**MAGNETIZATION TRANSFER (MT)**		
Images with (M_T_) and without (M_0_) MT pulse	MT ratio (MTR) (MTR = (M_0_ – M_T_)/M_0_)	Degree of myelination, axonal density
**MAGNETIC RESONANCE SPECTROSCOPY**		
^1^H	N-acetyl-aspartate (NAA) Creatine (Cr) Choline (Cho) Myo-Inositol (mIns) Glutamate/Glutamine, GABA	Neuronal number and health Used as reference concentration Demyelination and cell proliferation Osmotic stress or astrogliosis Neurotransmitters
^31^P	ADP/ATP/PCr	Energy metabolism
**RESTING-STATE FUNCTIONAL MRI**		
Blood oxygen level dependent (BOLD) contrast	Temporal correlation of BOLD signal fluctuations	Functional connectivity within brain networks
**ARTERIAL SPIN LABELING (ASL)**		
ASL perfusion imaging	Cerebral blood flow	Brain perfusion

a *The Magnetic Resonance Parkinsonism Index (MRPI) is defined by the product of the pons to midbrain area ratio (P/M) by the middle to superior cerebellar peduncles width ratio (MCP/SCP)*.

b *The MRPI 2.0 is defined by the product of the MRPI by the third ventricle width/frontal horns width ratio (V3/VL)*.

## Imaging Findings in Parkinsonian Disorders

### Parkinson's Disease

Parkinson's disease is an alpha-synucleinopathy, the main neuropathological characteristic of which is the neurodegeneration of the dopaminergic neurons of the substantia nigra *pars compacta* (SNpc) (1–3, 15, 16). In addition to SN degeneration, several other nuclei in the brainstem, basal forebrain and cortex are affected in PD in later stages, which helps to explain the presence of non-motor symptoms (15, 16). However, conventional MRI most often does not reveal specific abnormalities in PD outside the SN, and the basal ganglia are normal or only show subtle changes in terms of volume, diffusion measurements or iron deposition (8, 17–19). Lesions of the small brainstem nuclei, basal forebrain and cortex are mostly detected using specific quantitative approaches that are not used in clinical practice except for the locus coeruleus (18).

#### Conventional and Structural Imaging

##### Substantia nigra

**Figure 1:**
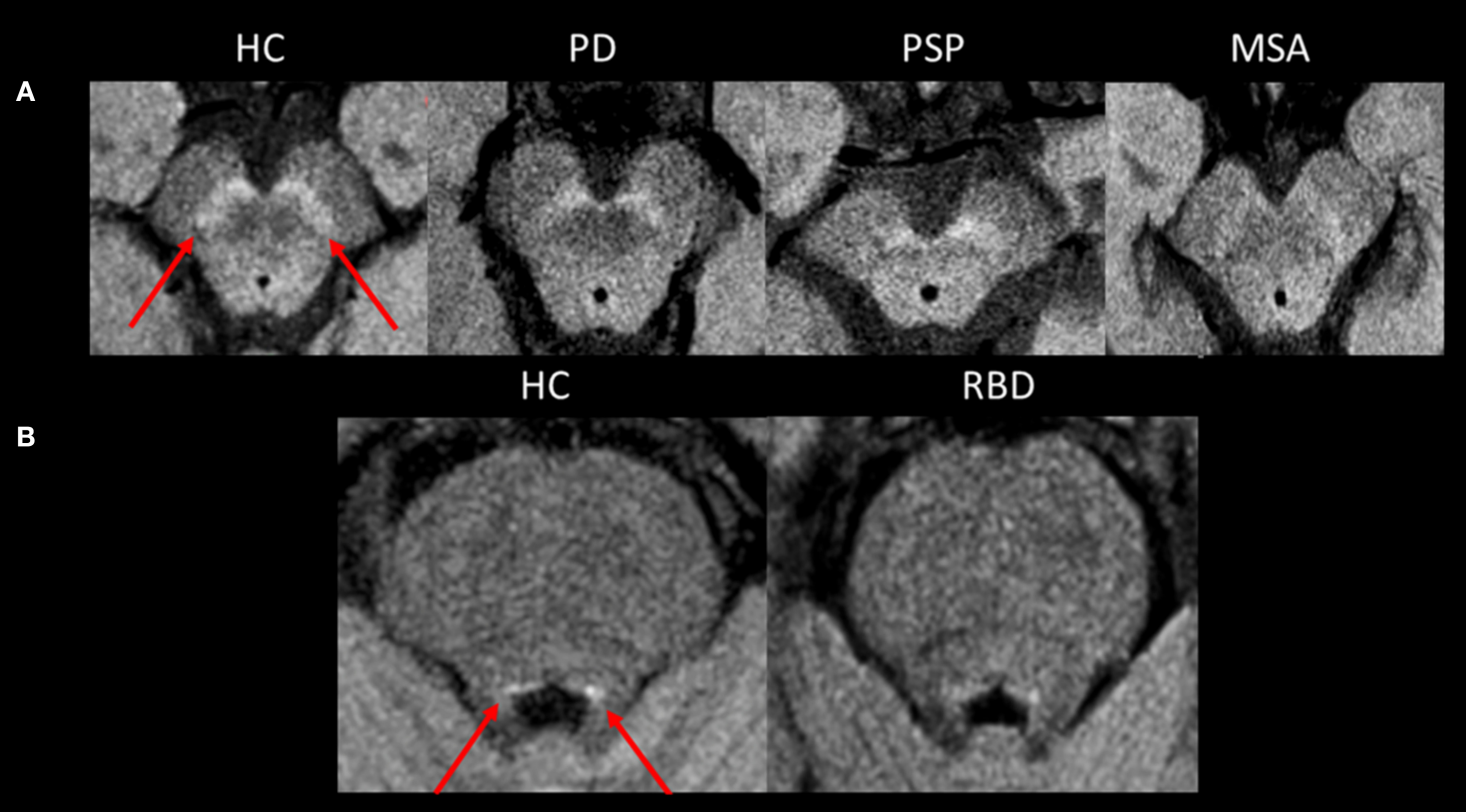
Neuromelanin imaging. **(A)** Axial neuromelanin-sensitive images at 3 Tesla passing through the midbrain at the level of the substantia nigra *pars compacta* in a healthy control and patients with PD, PSP, and MSA. The substantia nigra *pars compacta* is seen as an area of high signal in a healthy control (arrows). Its size and signal are decreased in neurodegenerative Parkinsonian syndromes. **(B)** Axial neuromelanin-sensitive images at 3 Tesla passing through the midbrain at the level of the coeruleus/subcoeruleus complex. The signal within the coeruleus/subcoeruleus complex is decreased in the patient with RBD compared to the healthy control. HC, healthy control; MSA, multiple system atrophy; PD, Parkinson's disease; PSP, progressive supranuclear palsy; RBD, rapid-eye movement sleep behavior disorder.

In clinical practice, degeneration of the SNpc can be detected by visual inspection of the images using neuromelanin-sensitive and iron-sensitive imaging. These nigral changes differentiate neurodegenerative Parkinsonism from essential tremor and other non-degenerative Parkinsonian syndromes (20). Neuromelanin-sensitive imaging relies on the T1 shortening effect of neuromelanin, a pigment contained in the SNpc, that has paramagnetic properties when it is bound to iron (17, 21, 22). A direct correlation between the signal intensity of post-mortem samples of SNpc on neuromelanin-sensitive sequences and the density of neuromelanin-containing neurons has been demonstrated (23). Using neuromelanin-sensitive imaging, the SNpc appears as an area of high signal intensity in healthy subjects. Patients with degenerative Parkinsonian syndromes show a reduction in the size and signal intensity of the SNpc, reflecting the loss of dopaminergic neurons (11, 24, 25) (Figure 1). The sensitivity and specificity of this technique are above 80%, and the performances between the visual assessment of trained radiologists and the quantification of volume and signal intensity are similar (26). In line with neuropathological findings (7), greater involvement of the posterolateral part of the SNpc with relative preservation of the medial part has been shown in patients with PD (27). A neuromelanin signal decrease has also been observed in patients with idiopathic rapid eye movement sleep behavior disorder (RBD) (28). RBD is a frequent non-motor feature of PD characterized by abnormal behavior and increased muscle tone during rapid eye movement sleep (29, 30). Patients with RBD have a high risk of developing Parkinsonism, including PD, dementia with Lewy bodies and, more rarely, MSA, with a rate of conversion of 33.5% within 5 years and 82.4% at 10 years, as reported by a recent meta-analysis (31). Idiopathic RBD is therefore considered a prodromal phase of Parkinsonism and PD, with an estimated period of 10–15 years of progressive neuronal loss before the onset of the main motor symptoms (31–33).

**Figure 2:**
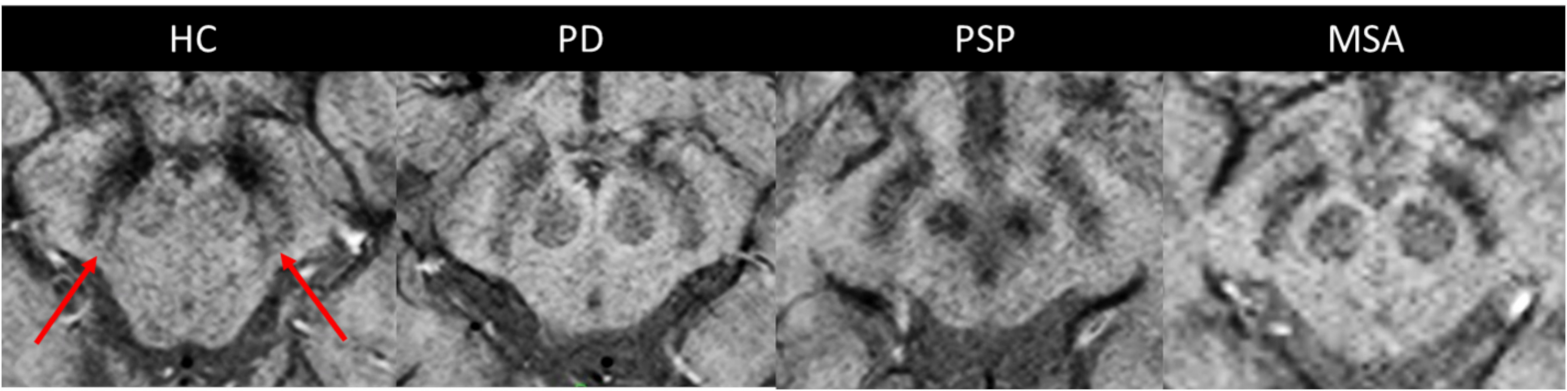
The dorsolateral nigral hyperintensity. Axial susceptibility-weighted images at 3 Tesla passing through the midbrain at the level of the substantia nigra *pars compacta* in a healthy control and patients with PD, PSP, and MSA. The dorsolateral nigral hyperintensity is well-depicted at the dorsolateral part of the substantia nigra *pars compacta* in a healthy control (arrows). It is lost in neurodegenerative parkinsonian syndromes. HC, healthy control; MSA, multiple system atrophy; PD, Parkinson's disease; PSP, progressive supranuclear palsy.

Using susceptibility-weighted imaging (SWI), healthy subjects show an area of normal high signal intensity located at the dorsolateral part of the SNpc, a feature also known as the “swallow tail sign” or the “dorsolateral nigral hyperintensity” (DNH) sign (34). Histopathological correlations have shown that the DNH corresponds to nigrosome 1, a region where dopaminergic neurons are affected early and severely (35) (Figure 2). The loss of this hyperintensity in Parkinsonian subjects is probably due to the presence of iron deposits in Parkinsonian subjects (17, 22). The absence of DNH was shown to be predictive for ipsilateral dopamine transporter (DAT) deficiency on radiotracer imaging, with high sensitivity and specificity (87.5 and 83.6%), supporting its potential as a marker of SN pathology (36). This DNH sign has a sensitivity between 79 and 100% and a specificity between 85 and 100% for the differentiation of degenerative Parkinsonian syndromes from healthy subjects (34, 36, 37). The DNH sign is also an early marker observed in two-thirds of idiopathic RBD patients (38).

##### Locus coeruleus/subcoeruleus complex

The locus coeruleus/subcoeruleus complex is composed of catecholaminergic neurons containing neuromelanin and is affected in approximately two-thirds of PD patients (39, 40). Using neuromelanin-sensitive imaging, the signal is decreased within the coeruleus/subcoeruleus complex of these patients. The involvement of this nucleus has been associated with the presence of RBD in PD patients and with idiopathic RBD (40, 41). The relationship between RBD and signal changes in this complex suggests that RBD in PD may be related to damage of the subcoeruleus part of the complex that is involved in the control of movement during REM sleep (29, 30, 40).

##### Brainstem

Structural brain imaging using conventional MRI with visual assessment of T2- and T1-weighted sequences is usually normal in patients with PD. Its main role is to detect or exclude secondary causes of Parkinsonism (such as vascular encephalopathy, demyelinating lesions, tumors or normal pressure hydrocephalus) and to look for signs of atypical Parkinsonism (10, 13, 19, 42). Manual morphometric measurements of the brainstem, including the anteroposterior midbrain diameter (43, 44), the midsagittal midbrain area, the midbrain to pons area ratio (43, 45) and the magnetic resonance Parkinsonism index (MRPI), are normal in PD (45, 46). Atrophy rates of the brainstem are not different from those of healthy subjects (47).

##### Other areas

Overall, except for the SN, the basal ganglia are mostly spared in PD patients (48, 49). In the cortex, no or mild changes have been reported in early PD (50). Conversely, impaired cognition and dementia in PD have been associated with marked cortical atrophy in many brain regions, including the frontal, parietal, and temporal areas, and the substantia innominata (50–52). Cortical atrophy accelerates with disease progression and worsening of cognitive decline (50). Longitudinal MRI studies have reported a higher rate of cortical thinning in PD patients with mild cognitive impairment than in PD patients with normal cognition and healthy subjects, which is correlated with cognitive decline (50, 51).

#### Diffusion Imaging

In the SN, changes in diffusion metrics have been reported at the group level. Using diffusion tensor imaging (DTI), reduced fractional anisotropy (FA) (26, 53) and increased diffusivity (26) values were evidenced in the SN in PD patients. However, there is a large variability of results across studies, and these measures do not appear useful at the individual level (54). Using DTI, two meta-analyses reported either frontal predominance of extranigral FA changes (55) or more widespread FA and mean diffusivity (MD) changes involving the corpus callosum and cingulate and temporal cortices in PD patients (56). Most studies did not detect any significant changes using global region-of-interest analyses in the putamen (57–61), midbrain (62, 63), pons (57–61, 64) and cerebellum (65). Free water is a more advanced diffusion metric derived from a bi-tensor diffusion model that shows better sensitivity and reliability for the discrimination of PD from healthy subjects. Increased free water values have only been observed in the SN in PD (66, 67), while no changes were found in other brain regions (66). In contrast, using region-of-interest measurements, diffusivity changes were measured in specific regions that are affected in PD, including the pedunculopontine nucleus in relation to gait disorders (68), the locus coeruleus for RBD (40), the medulla oblongata for autonomic dysfunction (69), and basal forebrain for cognitive decline (70).

#### Iron Imaging

Using R2^*^ relaxometry (71, 72) or quantitative susceptibility mapping (QSM) (71, 73, 74), an increase in iron deposition was detected in the SN, with QSM showing greater accuracy than R2^*^ relaxometry (71, 75, 76). Outside the SN, R2^*^, and susceptibility values were mostly normal in the putamen (61, 73, 74, 77). However, an increase in susceptibility values was reported in the red nucleus and globus pallidus of patients with late-stage PD (73).

#### Summary

The neuromelanin signal decrease within the SNpc is an early marker of Parkinsonism, useful at the individual level, meeting the criteria for a level 3 biomarker. Neuromelanin signal also correlates with the reduction of dopaminergic neurons in the SN and may be a surrogate marker of dopaminergic neuron degeneration (level 4). The DNH sign is also a level 3 biomarker and may hold potential for being a level 4 biomarker, although a direct correlation with the SN degeneration has not yet been evidenced. Neuromelanin signal decrease within the coeruleus/subcoeruleus complex may also be used as an early biomarker of Parkinsonism at the individual level (level 3). Free water changes appear useful at the individual level (level 2) although their correlation with neurodegenerative changes remains to be determined. Their use in clinical practice would require normative values that are currently not available in Radiology departments. Other findings were mostly shown at the group-level (level 1), with no demonstrable clinical relevance to date.

### Progressive Supranuclear Palsy

Progressive supranuclear palsy (PSP) is a tauopathy characterized by the presence of intracerebral depositions of Tau protein and neurodegeneration predominant in the brainstem, basal ganglia and cerebellar nuclei (3, 5, 6, 78, 79). PSP has a heterogeneous clinical expression. The most common clinical presentation of PSP is Richardson's syndrome (PSP-RS), which is characterized by early axial rigidity, postural instability, falls and vertical supranuclear gaze palsy (5, 79, 80). There are a number of other clinical phenotypes of PSP, including PSP with predominant Parkinsonism (PSP-P), progressive gait freezing (PSP-PGF), postural instability (PSP-PI), frontal presentation (PSP-F), speech/language disorder (PSP-SL), ocular motor dysfunction and corticobasal syndrome (PSP-CBS) (5, 79, 80). Imaging patterns in PSP variants are not clearly understood since most studies have focused on PSP-RS and/or did not differentiate PSP variants.

#### Conventional and Structural Imaging

##### Substantia nigra

As in PD, PSP patients also show a reduction in the neuromelanin signal and volume of the SNpc (27, 81, 82) and a loss of the DNH sign (37). Neuropathological studies have reported a different pattern of nigral degeneration from PD without predilection for the lateral part (7). Only one study using neuromelanin-sensitive imaging reported a difference between signal changes in PD and PSP (27), but this requires further investigation.

##### Locus coeruleus/subcoeruleus complex

The coeruleus/subcoeruleus complex is also involved in PSP (3, 5, 6). In line with histological studies, decreased neuromelanin signal of the coeruleus/subcoeruleus complex has been reported in PSP (18), but other studies did not find such changes (27).

##### Brainstem

**Figure 3:**
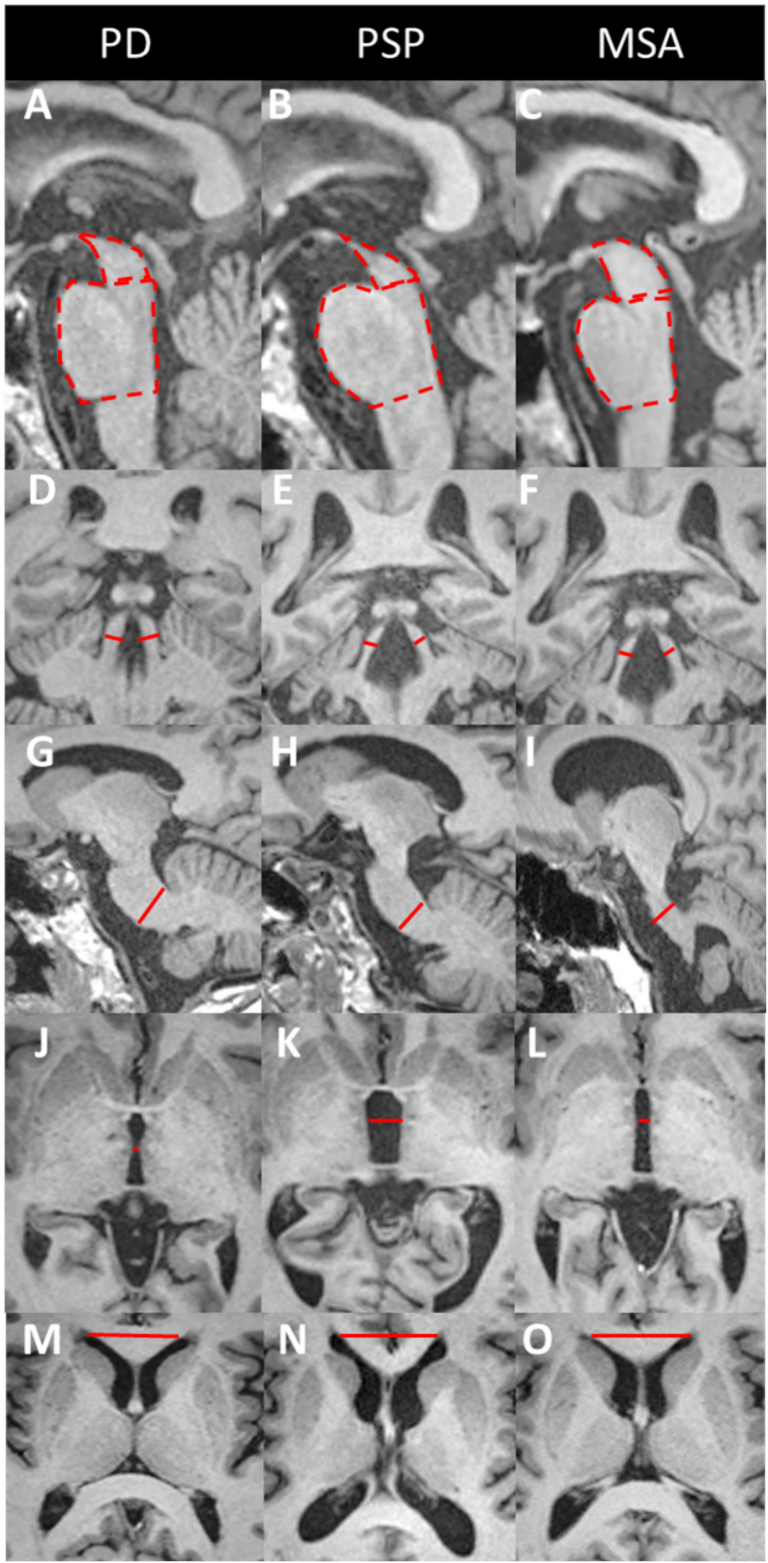
Midbrain to pons area ratio, Magnetic resonance Parkinsonism index (MRPI) and MRPI 2.0. Three-dimensional T1-weighted images in patients with PD (left), PSP (middle), and MSA (right). The MRPI combines measurements of the midsagittal surfaces of the midbrain (dashed line) and pons (dashed line) **(A–C)** and widths of the superior (red lines, **D–F**) and middle (red line, **G–I**) cerebellar peduncles [MRPI = (pons/midbrain) × (middle cerebellar peduncle/superior cerebellar peduncle)]. The limit between the midbrain and pons is defined by the line passing through the inferior border of the inferior colliculus and the pontomesencephalic sulcus. The inferior border of the pons is parallel to the previous one. The widths of the superior **(D–F)** and middle cerebellar peduncles **(G–I)** are measured in the parasagittal and coronal planes that best show the peduncles, respectively. The MRPI 2.0 is the product of the MRPI by the ratio of the width of the third ventricle (red line, **J–L**) to the largest left-to right width of the frontal horns of the lateral ventricles (red line, **M–O**) on axial views. For further details about the measurement technique [please refer to Quattrone et al. (46, 85)]. PSP patients show atrophy of the midbrain **(B)** and superior cerebellar peduncles **(E)** and enlargement of the third ventricle **(K)**. In MSA patients, the atrophy is greater in the pons **(C)** and middle cerebellar peduncles (I), with an enlarged fourth ventricle. PD patients do not show any significant brainstem atrophy or MRPI changes. MSA, multiple system atrophy; PD, Parkinson's disease; PSP, progressive supranuclear palsy.

**Figure 4:**
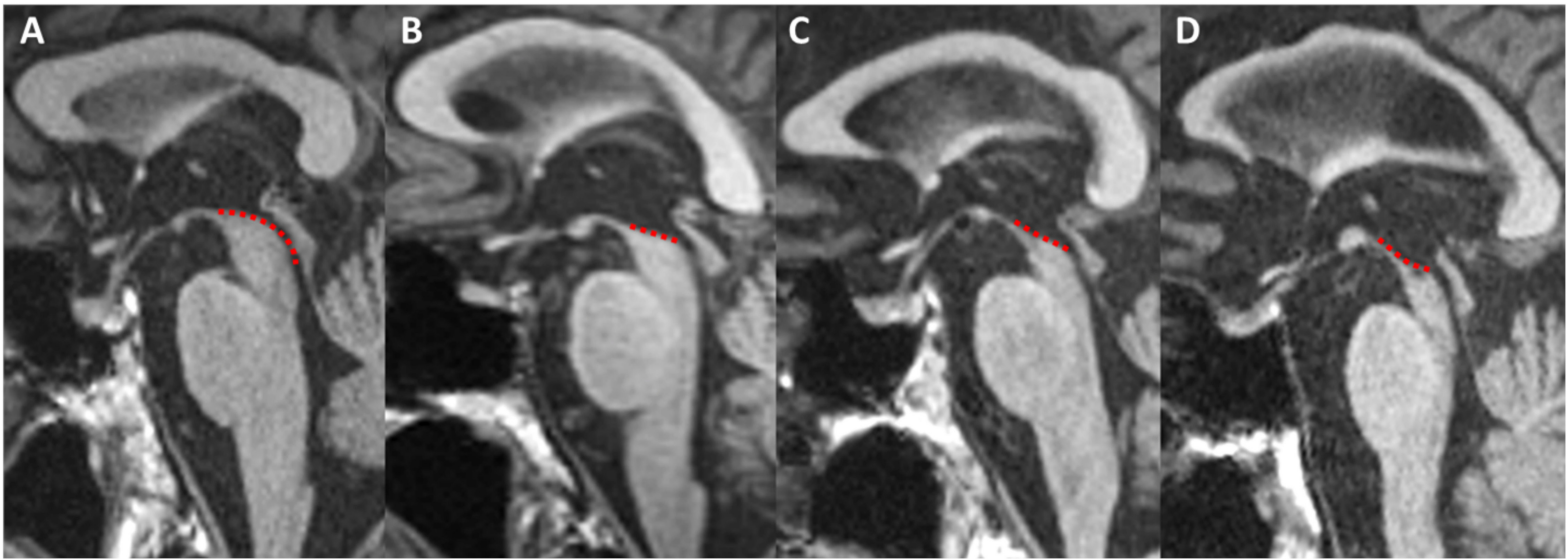
Midbrain atrophy in PSP patients. Midsagittal T1-weighted images in a healthy control **(A)** and PSP patients with increasing midbrain atrophy **(B–D)**. Healthy subjects and PD patients show a normal convex profile of the midbrain superior surface. PSP patients present a flat (mild) or concave (moderate to severe) aspect (dashed line). This shape is known as the “penguin” or “hummingbird” sign. HC, healthy control; PD, Parkinson's disease; PSP, progressive supranuclear palsy.

Patients with PSP-RS typically exhibit marked midbrain atrophy. The “hummingbird” sign (83), less frequently called the “penguin” sign (84), describes the flat or concave aspect of the midbrain tegmentum visible on midline T1-weighted sagittal sections (Figures 3, 4). The “morning glory” sign corresponds to the concave aspect of the lateral margin of the midbrain tegmentum on axial slices, which is also observed in PSP-RS (86). Although these features are suggestive of PSP-RS with a specificity of 99.5% for the “hummingbird sign” and 97.7% for the “morning glory sign,” they show low sensitivity (51.6 and 36.8%, respectively) (87) (Table 2).

**Table 2:** Magnetic resonance findings in progressive supranuclear palsy.

**Imaging biomarker**	**Findings**	**Performance (sensitivity/specificity, AUC)**	**References**
**MIDBRAIN**			
Hummingbird/penguin sign	Flat or concave superior profile in sagittal images	51.6/88.8%	Mueller et al. (87)
		68/88.8%	Righini et al. (44)
Morning glory sign	Concavity of the lateral margin of the midbrain on axial images	36.8/97.7%	Mueller et al. (87)
Anteroposterior diameter	<8.9 vs. PD and MSA	90/90%, AUC 0.94	Mangesius et al. (43)
	<12 mm vs. PD		Righini et al. (44)
Sagittal area	<122 mm^2^ vs. PD	83/84%	Moller et al. (45)
	<117 mm^2^ vs. MSA-P	76/82%	
	<114 mm^2^ vs. MSA-C	74/81%	
Midbrain/pons ratio	<0.18 vs. PD and MSA	81/87%	Mangesius et al. (43)
	<0.215 vs. PD and MSA-P	76.4/80%	Moller et al. (45)
	<0.275 vs. MSA-C	96.2/81%	Moller et al. (45)
MRPI	>13.6 vs. PD and HC	100/100%	Quattrone et al. (46)
	>12.9 vs. MSA-P	100/100%	Quattrone et al. (46)
MRPI 2.0	>2.5 vs. PD and HC	100/100%	Quattrone et al. (85)
FLAIR signal	Increased vs. PD	28/100%	Righini et al. (44)
Diffusivity	Increased vs. PD and MSA-P	81/81%, AUC: 0.90	Surova et al. (62)
			Tsukamoto et al. (63)
**SUPERIOR CEREBELLAR PEDUNCLES**			
Volume	Reduced vs. PD and MSA	74/94%	Paviour et al. (88)
			Quattrone et al. (46)
FLAIR signal	Increased	19.6/100%	Kataoka et al. (89)
Diffusivity	Increased		
	• vs. PD	100/100%	Nicoletti et al. (90)
	• vs. MSA-P	96.4/93.3%	
**PUTAMEN**			
Volume	Reduced vs. PD	AUC: 0.93	Pyatigorskaya et al. (18)
			Messina et al. (48)
Diffusivity	Increased vs. PD	75/100%	Nicoletti et al. (58)
		90/100%	Schocke et al. (91)
Susceptibility, R2*	Increased vs. PD	AUC: 0.83	Sjöstrom et al. (74)
			Lee et al. (77)
**THALAMUS**			
Volume	Decreased vs. PD and MSA-P	AUC: 0.86	Pyatigorskaya et al. (18)
			Messina et al. (48)
			Surova et al. (62)
Diffusivity	Increased vs. PD and MSA-P	81/77%, AUC: 0.81	Surova et al. (62)
**GLOBUS PALLIDUS**			
Volume	Decreased vs. PD and MSA-P	AUC: 0.86	Lee et al. (77)
			Messina et al. (48)
			Surova et al. (62)
Diffusivity	Increased	——–	Nicoletti et al. (58)
			Seppi et al. (92)
			Schocke et al. (91)
Susceptibility	Increased		Sjöstrom et al. (74)
	• vs. PD	AUC: 0.75	
	• vs. MSA	AUC: 0.73	
**RED NUCLEUS**			
Susceptibility	Increased		Sjöstrom et al. (74)
	• vs. PD	AUC: 0.97	
	• vs. MSA	AUC: 0.75	
**CORTICAL REGIONS**			
Frontal cortex	Atrophic	——–	Worker et al. (93)
			Huppertz et al. (94)

*The table provides optimal cut-off points with the corresponding sensitivity and specificity, as proposed by the studies. AUC values were reported when available. These values depend on which disease groups are compared. They were not validated and may vary from one study to another. The patient characteristics for each reference are listed in the Supplementary Table*.

*AUC, area under the ROC curve; DP, proton density-weighted; HC, healthy controls; MRPI, Magnetic Resonance Parkinsonism Index; MSA, multiple system atrophy; MSA-C, cerebellar variant of MSA; MSA-P, Parkinsonian variant of MSA; PD, Parkinson's disease; PSP-RS, progressive supranuclear palsy with Richardson syndrome; SWI, susceptibility-weighted imaging*.

Several manual morphometric indexes have been proposed as clinical biomarkers usable in individual patients. The anteroposterior midbrain diameter has limited discriminating power with a significant overlap of individual values (9, 44). The midbrain midsagittal area discriminate PSP-RS from healthy subjects and PD patients (<122 mm^2^) with sensitivity and specificity above 80% and from MSA patients (<114 mm^2^) with a lower sensitivity (45). The midbrain to pons midsagittal surface ratio (<0.18) has a sensitivity and specificity >90% in differentiating PSP-RS from healthy subjects, PD and MSA-P patients (43, 46), although some studies have found lower accuracies (45).

The superior cerebellar peduncles (SCPs) are atrophic in PSP, while the middle cerebellar peduncles (MCPs) are relatively spared, resulting in an increase in the MRPI in PSP. Compared to the midbrain to pons ratio, the MRPI showed better performance for the differentiation of PSP-RS from PD (when > 13.6) and from MSA-P (when > 12.9), with very good (43) to excellent sensitivity and specificity (46, 95, 96) (Figure 3, Table 2). Manual and automated approaches have shown similar performance for measuring the MRPI (96). A recent study has validated the use of a web platform providing an automated calculation of the MRPI, suggesting that the approach could be applicable in clinical practice (97). Automated MRPI values showed high performance in differentiating PSP-RS and PSP-P patients from non-PSP participants (93.6 and 86.5% accuracy, respectively). Differentiation was also good at the early stage of the disease (90.1 and 85.9%, respectively) (97). More recently, the MRPI 2.0 has been introduced to improve the discrimination of PSP-P from PD over that of the MRPI (sensitivity: 100% for the MRPI 2.0 vs. 73.5% for the MRPI) (85). Abnormal MRPI and MRPI 2.0 values also appeared to be early features of PSP-P (98) (Figure 3, Table 2).

In line with studies based on morphometric measurements, group-level studies using voxel-by-voxel analyses (49, 94, 99) or automated segmentation software, such as Freesurfer (48, 100) have reported volume reductions in the brainstem, including the midbrain and superior cerebellar peduncles, in PSP-RS.

Longitudinal studies have shown that the rates of midbrain atrophy in PSP (2.2 ± 1.5% per year) were seven times greater than those in healthy subjects (47). Furthermore, motor deficit severity correlates with midbrain atrophy (47). Similarly, the rate of progression of brainstem atrophy measured by the MRPI and MRPI 2.0 over 1- and 2-years intervals was higher in PSP patients than in PD patients (101). The progression rate was greater in PSP-RS patients than in PSP-P patients (101).

There is evidence that midbrain atrophy is a marker of PSP-RS phenotype rather than PSP pathology, as suggested by the absence of midbrain atrophy in patients with autopsy-proven PSP without clinical presentation of PSP-RS (9, 102). Other studies have also found midbrain atrophy in PSP variants, including PSP-P (9, 46, 98, 103), PSP-SL (102, 104, 105), and PSP-F (9, 102, 106), although it is typically less severe in these variants than in PSP-RS (9, 103, 107).

##### Other regions

In PSP-RS, among the basal ganglia, the volumes of the putamen and globus pallidus are smaller than those in PD, and the volume of the thalamus is smaller than that in MSA-P (8, 9, 48, 62). The usefulness of these findings at the individual level remains to be determined (9). Cortical atrophy with preferential frontal involvement has been demonstrated in PSP-RS patients but not in healthy subjects, PD and MSA patients at the group level (18, 93, 94). Frontal atrophy also occurs in PSP variants, particularly in PSP-F (9, 106), PSP-SL (9, 108), and PSP-CBS (9, 109), and to a greater extent than that of PSP-RS, probably reflecting different pathological loads between the brainstem and the cortex (9). The rate of frontal atrophy in PSP was shown to be three times that of healthy subjects and twice that of PD (47). Executive dysfunction correlated with increased rates of frontal atrophy in PSP (47).

#### Diffusion Imaging

In the SN, decreased FA (18) and increased free water (66) have been reported at the group level in PSP. Diffusion-weighted imaging (DWI) studies using manual measurements of apparent diffusion coefficient (ADC) and DTI studies in PSP-RS patients have demonstrated increased diffusivity values in the putamen, the caudate nucleus and the globus pallidus compared with those in PD patients and healthy subjects (58, 63, 92, 110, 111), as well as in the thalamus (58), the midbrain (18, 63, 110), the SCP (90, 110, 112), and the precentral and prefrontal white matter (58, 110) compared with those in PD, MSA and healthy subjects (58, 110, 111). In the SCP, ADC values were higher in PSP-RS than in healthy subjects and PD patients with a sensitivity of 90–100% and a specificity of 85–100% (90, 111) and in MSA-P patients with a sensitivity of 96.4% and a specificity of 93.3% (90), supporting the use of this measurement as a level 2 biomarker (9). Putamen diffusivity values were higher in PSP than in PD but overlapped between PSP and MSA-P (57, 90, 92, 113). Diffusivity in the thalamus was greater in PSP patients than in MSA patients (62) (Table 2). Reduced FA has been reported in the midbrain, pons, SCP, thalamus, cerebellar white and gray matter, dentate nucleus, corpus callosum, the precentral, superior frontal and parieto-occipital gray matter and the precentral white matter of PSP patients compared to healthy subjects and PD patients (18). In line with these results, free-water measurements were increased in the basal ganglia, midbrain, thalamus, dentate nucleus, cerebellar peduncles, cerebellar vermis and lobules V and VI, and corpus callosum in PSP patients compared with those in PD patients and healthy subjects (66). Free water-corrected fractional anisotropy values were increased in the putamen, caudate, thalamus, and vermis and decreased in the superior cerebellar peduncle and corpus callosum (66).

Tractography studies have shown more specific involvement of the SCP, corpus callosum and superior longitudinal fasciculus in PSP than in PD and MSA-P (9).

#### Iron-Sensitive Imaging

PSP patients show increased iron deposition in many regions. R2^*^ relaxation rates were increased in the putamen, caudate nucleus and globus pallidus compared to those in PD patients and healthy subjects (77, 114). R2^*^ and QSM values in the putamen overlapped between PSP and MSA (74, 113). Using QSM, PSP patients had higher susceptibilities in the basal ganglia and thalamus than healthy subjects and early-stage PD patients (115) and in the red nucleus than in MSA patients (74) (Table 2).

#### Other Biomarkers

Other techniques have been investigated in a research setting, with differences only being observed at the group level. Compared to that in healthy subjects, the magnetization transfer ratio in PSP patients was reduced in the globus pallidus, putamen, caudate nucleus, SN, and white matter, possibly reflecting changes in the degree of myelination or axonal density in the affected structures (116). Using spectroscopy, metabolic changes were observed in the basal ganglia and the frontal areas. Reduced N-acetylcholine (NAA)/creatine (Cr) or NAA/choline (Cho) ratios were reported in the lentiform nucleus (117–121), and a reduced NAA/Cr ratio was reported in the frontal cortex (117). Using resting-state fMRI, PSP patients have shown connectivity disruptions between the dorsal midbrain tegmentum and subcortical and cortical networks, including those involving the cerebellar, diencephalic, basal ganglia, and cortical regions (122), and between the thalamus and the striatum, supplementary motor area and cerebellum (123). These findings help to understand the pathophysiology of Parkinsonism but are of little use in discriminating diseases.

#### Summary

Measurements of midbrain atrophy including the midbrain to pons ratio, the MRPI and the MRPI 2.0 are the most robust biomarkers for clinical diagnosis of PSP-RS at the individual level (level 2). Frontal atrophy in addition to midbrain atrophy has also been reported as a level 2 biomarker although the degree of evidence appears lower. Further, the midbrain to pons ratio, the MRPI and the MRPI 2.0 have proven to be useful for the early diagnosis of PSP-RS (level 3), but their relevance at the prodromal stage of the disease remains unclear. Increased MRPI and the MRPI 2.0 also appear to be early features in PSP-P (98). Relevant biomarkers have not yet been validated for other PSP variants. Diffusivity in the SCP has shown accuracies above 90% for the differentiation of PSP-RS from PD and MSA, suggesting its clinical utility as a level 2 biomarker. However, normative values in healthy subjects and cut-off values still need to be defined.

### Multiple System Atrophy

Multiple system atrophy (MSA) is a synucleinopathy characterized by the accumulation of alpha-synuclein within glial cytoplasmic inclusions (3, 4, 78). MSA patients typically present with autonomic dysfunction with a variable degree of Parkinsonism, more predominantly in the Parkinsonian variant (MSA-P), and with cerebellar signs, more predominantly in the cerebellar variant (MSA-C). MSA-P is mainly associated with changes in the putamen, whereas MSA-C shows prominent involvement of the cerebellum, pons and MCP (3, 4, 8, 78). Striatonigral degeneration and olivopontocerebellar atrophy often overlap in MSA patients (3, 4), and a clinical and brain imaging continuum exists between both variants as the disease evolves (3, 4, 78, 124).

#### Conventional and Structural Imaging

##### Substantia nigra

Nigral abnormalities can be depicted using neuromelanin-sensitive imaging (27, 81, 82) and SWI (37). Neuropathological descriptions and neuromelanin-based imaging studies have shown a similar pattern of nigral degeneration between MSA-P and PD, with a greater involvement of the lateral part of the SNpc (7, 27).

##### Locus coeruleus/subcoeruleus complex

The locus coeruleus/subcoeruleus complex may be involved in MSA (3), resulting in signal loss on neuromelanin-sensitive imaging (27) that is less severe than that in PD patients (3, 27).

##### Basal ganglia, brainstem and cerebellum

**Figure 5:**
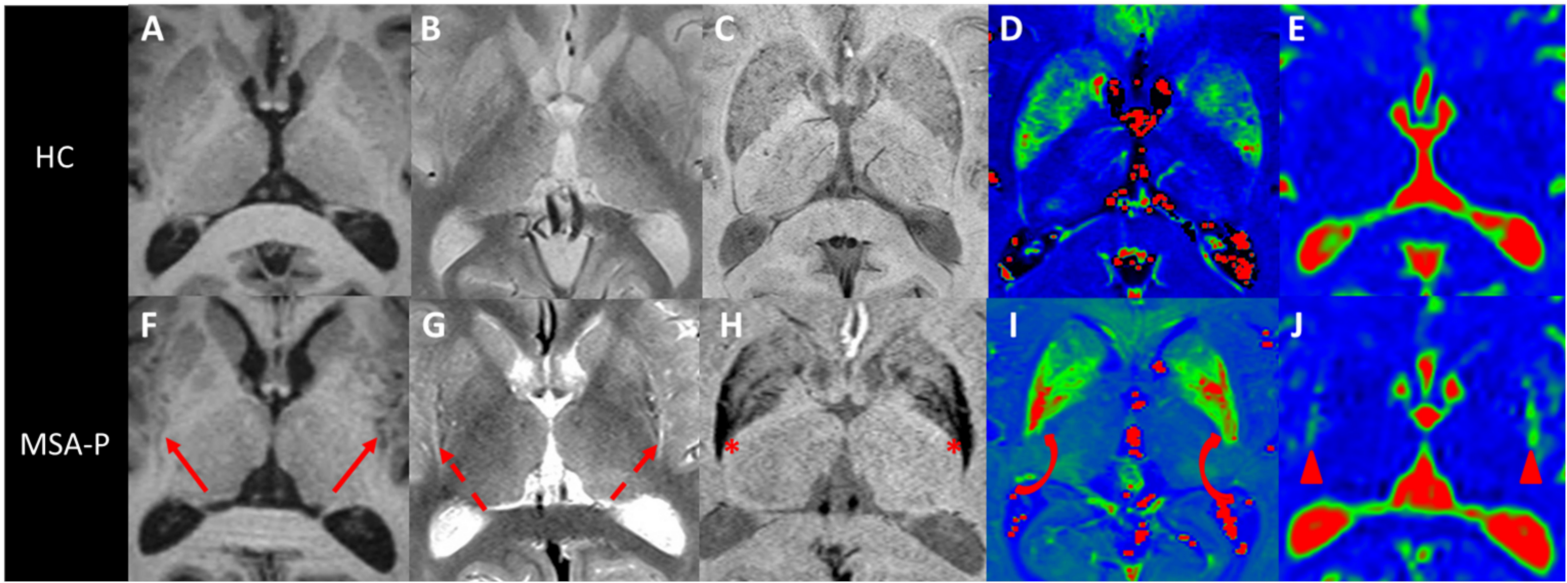
Putaminal abnormalities in MSA-P. Axial T1 **(A,F)**, proton density **(B,G)**, and susceptibility **(C,H)** weighted images and R2* **(D,I)** and ADC **(E,J)** maps passing through the putamen in a healthy control (upper row) and a patient with MSA-P (lower row). The posterior putamen are atrophic (**F**, arrows), showing a lateral hyperintense rim (**G**, dashed arrows), a hypointensity on susceptibility-weighted images (**H**, *) associated with increased R2* values (**I**, curved arrows), reflecting elevated iron depositions, and increased ADC values (**J**, arrowheads). ADC, apparent diffusion coefficient; HC, healthy control; MSA-P, Parkinsonian variant of multiple system atrophy; R2*, T2* relaxation rate.

In MSA-P, the putamen is atrophic, with a characteristic flattening of its lateral border, hypointense on T1-weighted gradient echo images, and hypointense in SWI due to the presence of iron deposits (125) (Figure 5). This hypointensity is surrounded by a rim of hyperintensity at its dorsolateral margin on proton density- and T2-weighted images (126) (Figure 5). In line with the underlying neuropathology (3), these signal changes are typically more prominent on the posterior part of the putamen (125). A hyperintense putaminal rim may occasionally be seen in PD patients and healthy subjects (3, 87, 95, 97). Signal abnormalities are influenced by the sequence parameters and the magnetic field strength of the MRI scanner (112). Hypointensity in the putamen have been shown to be greater using SWI than T2^*^- and T2-weighted images and increased with field strength (127). Therefore, overall sensitivity values are extremely variable across studies, ranging from 38 to 100% for T2 hypointensity in the putamen and from 56 to 90% for the hyperintense dorsolateral rim, whereas specificity values varied between 87 and 100% in distinguishing MSA-P patients from PD or healthy subjects (125) (Table 3).

**Table 3:** Magnetic resonance findings in multiple system atrophy.

**Imaging biomarker**	**Findings**	**Performance (sensitivity/specificity, AUC)**	**References**
**PONS**			
Sagittal area	MSA-P vs. PD <553 mm^2^	61.7/75.0%	Moller et al. (45)
	MSA-C vs. PD <538 mm^2^	81.0/82.8%	
Midbrain/pons ratio	MSA-P vs. PSP >0.215	76/80%	Moller et al. (45)
	MSA-C vs. PSP >0.275	96.0/81.2%	
	MSA-C vs. PD >0.290	81.0/79.9%	
MRPI	MSA-P vs. PSP <12.9	100/100%	Quattrone et al. (46)
Signal on PD-w images	Hot cross bun sign	——–	Way et al. (128)
Diffusivity	Increased	——–	Nicoletti et al. (58, 90)
			Pellecchia et al. (94, 99)
			Schocke et al. (91)
			Seppi et al. (92)
			Seppi et al. (129, 130)
			Barbagallo et al. (61)
			Chen et al. (64)
**MIDDLE CEREBELLAR PEDUNCLE**			
Volume	Decreased	——–	Quattrone et al. (46)
Signal on T2-w images	High signal	——–	Heim et al. (3)
Diffusivity	Increased	100/100%	Nicoletti et al. (58)
	MSA-P vs. PD and PSP		
**PUTAMEN**			
Volume	Reduced	——–	Seppi et al. (129, 130)
			Messina et al. (48)
			Scherfler et al. (100)
			Huppertz et al. (94)
Signal on SWI	Hypointensity	——–	Seppi et al. (129, 130)
Signal on T2/PD-w images	Hyperintense dorsolateral rim	——–	Seppi et al. (129, 130)
			Watanabe et al. (126)
Diffusivity	Increased		
	• vs. PD	90–100/93–100%	Bajaj et al. (72)
	• vs. PSP	100/81.2%	Nicoletti et al. (58)
			Pellecchia et al. (94, 99)
			Schocke et al. (91)
			Seppi et al. (92)
			Seppi et al. (129, 130)
			Barbagallo et al. (61)
R2*	Increased	77.8/100%	Focke et al. (113)
			Barbagallo et al. (61)
			Lee et al. (77)
Susceptibility	Increased vs. PD	AUC: 0.77	Sjöstrom et al. (74)
**CEREBELLUM**			
Volume	Decreased	——–	Scherfler et al. (100)
			Huppertz et al. (94)
Diffusion	Increased vs. PD and PSP	100/100%	Nicoletti et al. (65)

*AUC values were reported when available*.

*AUC, area under the ROC curve; DP, proton density-weighted; HC, healthy controls; MRPI, Magnetic Resonance Parkinsonism Index; MSA, multiple system atrophy; MSA-C, cerebellar variant of MSA; MSA-P, Parkinsonian variant of MSA; PD, Parkinson's disease; PSP-RS, progressive supranuclear palsy with Richardson syndrome; SWI, susceptibility-weighted imaging*.

**Figure 6:**
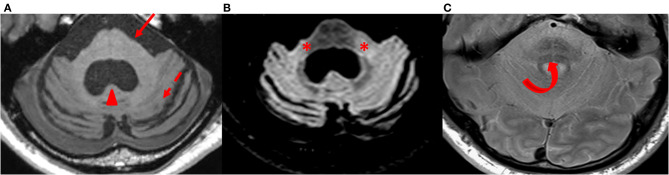
Findings in MSA-C patients. Axial T1-weighted **(A)**, FLAIR **(B)**, and proton density-weighted **(C)** images at the level of the pons and cerebellum in a patient with MSA-C. The pons (arrow) and the cerebellum (dashed arrow) are atrophic with an enlarged fourth ventricle (arrowhead) **(A)**. Middle cerebellar peduncles are hyperintense on FLAIR images (*) **(B)**. A “hot cross bun sign” is well visible in the pons (curved arrow) **(C)**. MSA-C, cerebellar variant of multiple system atrophy.

In MSA-C, the pons is atrophic, showing areas of increased signal intensity on proton density-weighted images affecting the transverse pontine fibers with the shape of a cross, well-known as the “hot cross bun” sign (126) (Figure 6). This sign has limited specificity, as it can be encountered in other pathologies, such as spinocerebellar ataxia (128). The MCPs present with atrophy and T2 hyperintensity, and the cerebellum is atrophic (8) (Table 3).

Regional brain atrophy can be quantified. While PSP patients have greater midbrain and SCP atrophy, MSA-C patients have predominant atrophy in the pons and MCPs, resulting in increased midbrain to pons ratio and decreased MRPI, with sensitivity and specificity equal to 95% and 100% for the categorization of MSA-P and PSP patients, respectively (46). Therefore, the midbrain to pons ratio and the MRPI could be suggested as level 2 biomarkers.

In MSA-P, atrophy rates have been shown to be greatest in the pons, equal to 4.5 ± 3.2% per year, over 20 times that in healthy subjects and three times the rate of pontine atrophy in PSP. Atrophy rates in the cerebellum (3.2 ± 1.9% per year) were more than ten times higher than those of healthy subjects and twice that of PSP (47). The severity of motor deficit correlated with ponto-cerebellar atrophy in MSA-P (47).

#### Diffusion

In MSA-P, diffusivity of the posterior putamen is increased, which is visible by simple inspection of ADC maps and can also be quantified (Figure 5). DWI studies have consistently reported an increase in ADC and Trace(D) values in the putamen in MSA-P compared to healthy subjects and PD patients (57–61, 90, 92, 129, 130), with an overall sensitivity of 90% and specificity of 93% in discriminating MSA-P from PD patients in a recent meta-analysis (57). These diffusion abnormalities were greater in the posterior putamen (59, 63, 129). UPDRS motor scores correlated positively with Trace(D) values in both the entire and posterior putamen in MSA-P patients (60). However, the ability of diffusivity measurements in the putamen to distinguish MSA and PSP is debated. Some studies have reported reliably higher ADC values in MSA-P than PSP (58, 111), while others have described a significant overlap of values, which would limit the interest for discriminating individual patients (57, 58, 129).

Other diffusion abnormalities in MSA-P involve the MCPs, the pons and the cerebellum (58–61, 64, 90–92, 130), reflecting a certain degree of olivopontocerebellar changes in addition to striatonigral degeneration (58). Increased ADC values in the MCPs allow complete differentiation of MSA-P from PD and PSP patients with 100% sensitivity and specificity (58). FA values are decreased in the MCP and pons (64). Diffusivity in the putamen and the MCP could therefore improve the categorization between MSA-P and MP on an individual basis. Increased diffusivity was also observed in the thalamus of MSA-P patients (58–61, 90–92) but to a lesser extent compared to PSP patients (62) (Table 3).

In MSA-C, the MCPs and cerebellum are more affected and the putamen is less affected than in MSA-P (8, 131, 132). Mean diffusivity in the cerebellum seems to be a robust discriminating marker, allowing the differentiation of MSA-P and MSA-C from PD and PSP-RS patients with a 100% positive predictive value (65) (Table 3).

Increased disease duration correlated with increased Trace(D) values in the pons of MSA-P patients and in the cerebellum and MCPs of MSA-C patients (64, 91).

#### Iron-Sensitive Imaging

MSA patients have greater iron deposits in the putamen than healthy subjects and PD patients (115), with greater involvement of the posterolateral part of the putamen (115, 133) (Figure 5). Increased R2^*^ values in the putamen showed 78% sensitivity and 100% specificity for the discrimination of MSA-P patients from healthy subjects and PD patients (61, 77, 113). R2^*^ values correlated positively with atrophy of the putamen (77). Similar differences are observed between MSA-C and PD (61). Using QSM, susceptibility values were also shown to be increased in MSA compared to PD (74). The combination of mean diffusivity and R2^*^ measurements in the putamen improved the distinction between MSA-P and PD patients, resulting in an accuracy of 96% (61). Although the signal decrease due to the presence of iron deposits observed using SWI in the putamen is visually greater in MSA-P than PSP, there is a significant overlap of R2^*^ and susceptibility values in the putamen between the two diseases (74, 113) (Table 3).

#### Other Biomarkers

The magnetization transfer ratio was reduced in the putamen of MSA patients (116). Using spectroscopy, decreased NAA/Cr and Cho/Cr ratios were reported in the lenticular nucleus in MSA-P compared to those in healthy subjects. The NAA/Cr ratio in the lenticular nucleus (118) and NAA/Cr in the cerebellum were decreased in MSA-C (134). The NAA/Cr ratio was decreased in the pons in both MSA types (135). Resting-state fMRI studies have shown connectivity abnormalities in the primary sensorimotor and premotor cortex and prefrontal, inferior parietal and occipital areas in MSA-C and MSA-P patients. Unlike in patients with PD, decreased or increased connectivity in different regions of the visual associative cortices and decreased connectivity in the right cerebellum were observed in MSA patients (136).

#### Summary

Although morphometric measurements, such as the midbrain to pons ratio and the MRPI were initially designed for PSP, these indices also allow differentiation MSA from PSP and PD. Thus, they could be used as clinical biomarkers for MSA (level 2). Other level 2 biomarkers include the characteristic shape of the putamen, T2^*^ and SWI signal decrease and ADC increase in the posterior putamen in MSA-P, and atrophy of the pons, cerebellar peduncles and cerebellum with the cross-bun sign in MSA-C. The potential utility of these measurements for the early diagnosis has to be investigated. Putamen abnormalities assessed by quantitative MRI techniques contribute to the diagnosis of MSA-P as level 2 biomarkers, although there is a significant overlap of diffusivity and R2^*^ values between PSP and MSA-P. Other potential clinical biomarkers include diffusivity in the MCP and the cerebellum.

## Machine-Learning-Based Differentiation of Parkinsonian Disorders

Very promising results have been obtained by combining fully automated quantitative MRI analysis with machine learning approaches to discriminate between Parkinsonian syndromes (94, 100, 137). Machine learning algorithms first learn to classify individual patients into different diagnostic categories using training data sets and then are applied to a new test data set. The algorithm then classifies each new subject in one of the groups of patients (138). Diagnostic precision is usually assessed with accuracy or balanced accuracy, which takes into account the differences in the number of subjects between the groups (94). A large multicenter study (*n* = 464) including healthy subjects (*n* = 73) and patients with PD (204), PSP-RS (109), MSA-P (20), and MSA-C (59) using a support vector machine (SVM) trained with MRI morphometric data reported balanced accuracies above 80% for between-group classification (94). The volumes of the midbrain, basal ganglia, and cerebellar peduncles had the largest contribution to group differentiation (94). Another volumetric study including 110 Parkinsonian patients in the early to moderate stages of the disease (40 PD, 40 MSA, 30 PSP) using measurements in 22 subcortical regions showed an accuracy of 97.4% for the discrimination of PD from to MSA and PSP (100). In contrast, the diagnostic accuracy using validated clinical consensus criteria obtained at the time of MRI acquisition was only 62.9%. The midbrain, putamen and cerebellar gray matter volumes were the most significant brain regions involved in this classification (100).

Combining volumetric measures with diffusion or iron measures could improve categorization. In one study, the combination of volumetry and DTI metrics allowed 100% accuracy in the discrimination of PD and PSP patients using SVM (99). Adding R2^*^ values to volumetry and DTI metrics resulted in 95% accuracy for the classification of PD, MSA-C and MSA-P patients in another study (139). Machine learning approaches appeared also applicable to data from different clinical departments and MRI scanners. A recent multicenter study used free water and free water-corrected fractional anisotropy measurements as input for a linear SVM algorithm to differentiate between Parkinsonian syndromes (140). This automated pipeline was referred to as automated imaging differentiation in Parkinsonism (AID-P). The study included the largest cohort of subjects to date (*n* = 1,002) collected from 17 international sites using MRI scanners from different vendors. Three models were tested, which included diffusion imaging measurements from several brain regions, scores from the Movement Disorders Society Unified Parkinson's Disease Rating Scale part III (MDS-UPDRS III), and both diffusion imaging and MDS-UPDRS III. In the differentiation of PD from atypical Parkinsonism, diffusion imaging plus MDS-UPDRS III showed a significantly higher AUC (0.962) than the MDS-UPDRS III score alone (0.775), similar to that of diffusion imaging alone (0.955). Similarly, diffusion imaging plus MDS-UPDRS III (AUC 0·897) and diffusion imaging alone (AUC 0.926) significantly outperformed MDS-UPDRS III alone (AUC 0.582) in the differentiation of MSA from PSP. Harmonization of diffusion imaging data did not significantly improve the performance of machine learning models. These fully automated approaches promise to be highly generalizable (140). Further studies including PSP variants are needed, however. In addition, validation as part of the diagnostic assessment of clinical populations would make it possible to implement machine learning approaches in clinical practice.

## Conclusion

Research using multimodal MRI in Parkinsonian syndromes has enabled the development of several *in vivo* biomarkers, some of which have demonstrated individual-level diagnostic utility. SNpc neurodegeneration in Parkinsonism is evidenced by a reduction in neuromelanin-sensitive signal and a loss of DNH in SWI. Similarly, the decrease in neuromelanin-sensitive signal in the coeruleus/subcoeruleus complex, which is associated with RBD, is an early marker of PD and MSA, and may be considered a surrogate biomarker for the degeneration of catecholaminergic neurons in this complex. However, to date, there is no definitive MRI biomarker of Parkinsonism. Overall, apart from SN changes and from the quantitative changes in specific regions of interest reported in group studies, conventional MRI used in clinical practice is generally normal in early PD patients. The midbrain to pons ratio and the MRPI are robust clinical biomarkers of PSP-RS, while abnormalities in the putamen (atrophy, flattening of the lateral border, T2, T2^*^ and SWI signal changes, and increased diffusivity) and in the pons and cerebellum (atrophy and signal changes) strongly suggest the diagnosis of MSA-P and MSA-C, respectively. Diffusion imaging and R2^*^ relaxometry allow accurate differentiation of groups, but the lack of normative and cut-off values, which vary with scanners, still hampers their use in clinical routine. New consensus criteria for the diagnosis of Parkinsonian syndromes incorporating MRI biomarkers should be considered in the future. Diagnostic accuracy could also benefit from machine learning approaches at a time when artificial intelligence promises to play a growing role in medicine.

## Author Contributions

LC drafted the manuscript. NP, BD, and DG revised the manuscript. SL drafted and revised the manuscript.

## Conflict of Interest

BD received research support grants from Fondation de France, Inserm, ANR; speech honoraria from Ipsen, Merz Pharma, Orkyn; and received travel funding from Merz Pharma, Elivie, Orkyn. SL received grants from Investissements d'avenir [grant numbers ANR-10-IAIHU-06 and ANR-11-INBS-0006] and Biogen Inc. The remaining authors declare that the research was conducted in the absence of any commercial or financial relationships that could be construed as a potential conflict of interest.
