# Supplementary material for: The Role of Magnetic Resonance Imaging for the Diagnosis of Atypical Parkinsonism
Source: Front Neurol. 2020 Jul 17;11:665. doi: 10.3389/fneur.2020.00665 (PMC7380089; doi:10.3389/fneur.2020.00665)
Supplement: Supplementary file 1 [file Table_1.pdf]

Supplementary table: Patient characteristics in the main studies referenced in Tables 2 and 3

Study	Groups	n	Gender (M/F)	Mean age ± SD	Mean disease duration ± SD
**Morphometry, Volumetry**					
Righini et al. 2004	PD	27	NA	67.6 ±3.7	5.3 ±3.4
	PSP	25		68.9 ±6.5	5.3 ±2.8
Quattrone et al. 2008	HC	50	25/25	65.8 ±9.0	
	PD	108	62/46	66.6 ±6.5	5.5 ±4.3
	PSP	33	23/10	69.3 ±6.1	3.0 ±1.6
	MSA-P	19	5/14	64.0 ±5.3	4.6 ±3.1
Messina et al. 2011	HC	46	20/26	66.78 ±6.7	NA
	PD	72	40/32	63.78 ±9.0	6.19 ±4.5
	PSP	32	21/11	70.63 ±5.3	3.53 ±3.5
	MSA-P	15	5/10	64.27 ±4.3	3.07 ±2.2
Huppertz et al. 2016	HC	73	37/36	NA	NA
	PD	204	136/68	56.9 ±0.9	6.5 ±0.4
	PSP-RS	106	60/46	66.1±0.6	3.2±0.2
	MSA-P	60	38/22	59.2±1.1	3.6±0.3
	MSA-C	21	11/10	59.5±1.8	3.8±0.5
Scherfler et al. 2016[1]	PD	40	6/8	61.7 ±7.3	3.3 ±2.2
	PSP	30	4/6	65.2 ±4.9	2.7 ±1.9
	MSA	40	11/3	62.7 ±7.8	2.9 ±1.5
Moller et al. 2017	HC	73	37/36	63.3 ±0.9	
	PD	204	136/68	64.0 ±0.8	6.5 ±0.4
	PSP	106	60/46	69.0 ±0.6	3.2 ±0.2
	MSA-P	21	13/8	63.3 ±1.0	3.6 ±0.3
	MSA-C	60	30/30	63.1 ±1.6	3.8 ±0.5
Mangesius et al. 2018[1]	PD	194	36/19	64.3± 9.2	2.4± 0.5
	PSP	55	10/7	66.8± 9.6	1.7± 0.6
	MSA	63	7/5	65.5± 7.3	2.1± 0.6
Quattrone et al. 2018	HC	53	36/17	71.5 ±5.2	NA
	PD	53	39/14	70.3 ±5.2	6.8 ±3.3
	PSP-RS	46	25/21	70.5 ±5.2	3.9 ±1.7
	PSP-P	32	27/7	72.0 ±5.7	7.3 ±3.7
Mueller et al. 2018	HC	79	42/37	67.2 ± 4.7	NA
	PD	289	183/106	66.2 ± 8.9	6.4 ± 6.2
	PSP	85	50/35	68.6 ± 9.4	2.8 ± 1.9
	MSA	97	47/50	63.4 ± 8.2	3.3 ± 2.4
**DWI, DTI**					
Seppi et al. 2003	PD	13	7/6	62 ±10.6	3.0 ±1.2
	PSP	10	5/5	63 ±6.6	2.9 ±1.1
	MSA-P	12	3/9	68 ±6.9	2.7 ±1.1
Schocke et al. 2004	HC	10	5/5	60 ±5.8	NA
	PD	17	9/8	62 ±8.0	3.7 ±1.8
	MSA-P	11	3/8	64 ±5.8	3.9 ±1.9

Nicoletti et al. 2006	HC<br>PD<br>PSP<br>MSA-P	15<br>16<br>16<br>16	5/10<br>9/7<br>13/3<br>4/12	67.5 ±6.0<br>61.0 ±7.7<br>70.7 ±7.8<br>64.7 ±5.1	NA<br>7.5 ±5.8<br>3.3 ±2.5<br>4.9 ±4.0
Seppi et al. 2006	HC<br>PD<br>MSA-P	11<br>20<br>15	NA	60 ±5.8<br>62 ±8.3<br>64 ±5.5	3.9 ±5.8<br>3.5 ±2.1
Seppi et al. 2006	PD<br>MSA-P	10<br>10	NA	64 ±5.5<br>66 ±10.9	4 ±2.3<br>5 ±2.2
Nicoletti et al. 2008	HC<br>PD<br>PSP<br>MSA-P	16<br>15<br>28<br>15	4/12<br>9/6<br>16/12<br>6/9	66.6 ±5.9<br>65.2 ±7.8<br>64.5 ±5.6<br>64.7 ±5.2	8.6 ±3.6<br>3.2 ±1.7<br>5.1 ±4.0
Pellecchia et al. 2009	HC<br>MSA-P<br>MSA-C	11<br>9<br>12	7/4<br>6/3<br>7/4	58.5 ±12.9<br>69.3 ±7.3<br>58.2 ±5.3	4.1 ±1.4<br>4.1 ±2.2
Pellecchia et al. 2011	MSA	11	5/6	63.6 ±10.1	3.4 ±1.1
Tsukamoto et al. 2012	HC<br>PD<br>PSP<br>MSA	18<br>17<br>20<br>25	NA	66.3 ±9.9<br>71.1 ±6.3<br>74.6 ±5.7<br>64.7 ±8.2	6.0 ±3.0<br>4.0 ±3.0<br>3.4 ±2.6
Nicoletti et al. 2013	HC<br>PD<br>PSP-RS<br>MSA-P<br>MSA-C	10<br>10<br>17<br>9<br>7	5/5<br>6/4<br>9/8<br>4/5<br>3/4	62.2 ±2.8<br>64.1 ±3.1<br>64.2 ±3.3<br>61.3 ±3.2<br>62.5 ±2.8	8.3 ±2.5<br>5.9 ±1.8<br>4.4 ±1.2<br>5.4 ±1.1
Surova et al. 2015[1]	HC<br>PD<br>PSP<br>MSA-P	21<br>10<br>27<br>11	12/9<br>6/4<br>14/13<br>4/7	69<br>68<br>68<br>64	4<br>3<br>3
Barbagallo et al. 2016	PD<br>MSA-P<br>MSA-C	26<br>16<br>13	12/14<br>7/9<br>6/7	63.8 ±6.3<br>66.1 ±7.8<br>61.5 ±6.5	7.4 ±4.5<br>5.4 ±2.2<br>6.1 ±2.5
Bajaj et al. 2017 [2]	HC<br>PD<br>MSA-P	60<br>262<br>127	NA	NA	NA
**Relaxometry, QSM**					
Lee et al. 2013	HC<br>PD<br>PSP<br>MSA-P	21<br>29<br>13<br>15	12/9<br>17/12<br>8/5<br>4/11	60.0 ±6.1<br>59.1 ±7.6<br>57.9 ±8.5<br>62.0 ±5.2	NA<br>2.5 ±1.9<br>1.9 ±0.7<br>2.3 ±1.3
Sjöstrom et al. 2017	HC<br>PD<br>PSP<br>MSA	14<br>62<br>15<br>11	9/5<br>43/19<br>12/3<br>6/5	63.5 ± 5.3<br>65.2 ±10.5<br>69.1 ± 6.0<br>68.9 ±13.1	NA<br>4.7 ±4.4<br>2.8 ±1.0<br>3.6 ±2.7
**Other**					
Kataoka et al. 2019	PSP	51	35/16	72.2 ±6.2	3.5 ±2.0

[1] Patient characteristics in the test cohort

[2] Metanalysis

Abbreviations: DWI, diffusion-weighted imaging; DTI, diffusion tensor imaging; HC, healthy controls; MSA, multiple system atrophy; MSA-C, cerebellar variant of MSA; MSA-P, parkinsonian variant of MSA; PD, Parkinson's disease; PSP, progressive supranuclear palsy
